# 1375. Strengthening Global Capacity to Detect Antimicrobial Resistance Threats

**DOI:** 10.1093/ofid/ofad500.1212

**Published:** 2023-11-27

**Authors:** Tina Benoit, Rachael Aubert, Jacob Clemente, Dawn Sievert

**Affiliations:** Centers for Disease Control and Prevention, Atlanta, Georgia; Centers for Disease Control and Prevention, Atlanta, Georgia; Centers for Disease Control and Prevention, Atlanta, Georgia; Centers for Disease Control and Prevention, Atlanta, Georgia

## Abstract

**Background:**

Antimicrobial resistance (AR) poses a major global threat, with a reported 1.27 million deaths in 2019. This resulted in years of life lost as well as a large burden on the healthcare system. Transmission of resistant pathogens is not limited to one geographic area and can easily be influenced by international travel and trade of food and other products. Addressing the global threat of AR is recognized as a major action item within the U.S. National Action Plan for Combating Antibiotic Resistant Bacteria. In 2021, the Centers for Disease Control and Prevention (CDC) established the Global Antimicrobial Resistance Laboratory and Response Network (Global AR Lab & Response Network). The network provides financial and technical support to partners, with a focus on detecting and monitoring existing threats, identifying risk factors, preventing and controlling spread, and mitigating the emergence of new threats.

**Methods:**

In just over 2 years, CDC has invested more than $40 million through the Global AR Lab & Response Network on projects with more than 20 organizations in nearly 50 countries. Using a multi-pathogen approach, the network focuses on healthcare-associated, sexually transmitted, fungal, enteric, and invasive bacterial and respiratory pathogens.

**Results:**

CDC’s Global AR Lab & Response Network is strengthening capacity to detect and respond to AR threats identified in the CDC 2019 AR Threats Report. Accomplishments include implementing trainings for high quality laboratory methods; monitoring resistance in bacteria and fungi to inform on changes in resistance and improve interventions; training and implementing activities to prevent, detect, and respond to AR in healthcare settings; and enhancing data sharing nationally, regionally, and globally.

**Conclusion:**

Through this innovative approach with plans for expansion, the Network will continue to use a One-Health methodology to focus on enhancing global capacity to quickly detect AR threats and built upon existing laboratory and response efforts. Findings from these projects will also inform intervention strategies to address current and future AR threats.

**Disclosures:**

**All Authors**: No reported disclosures

